# Treatment of baker cyst, by using open posterior cystectomy and supine arthroscopy on recalcitrant cases (103 knees)

**DOI:** 10.1186/s12891-016-1291-5

**Published:** 2016-10-18

**Authors:** M. Saylik, K. Gökkuş

**Affiliations:** 1 Orthopedics and Trauma Department, Attending orthopedic Surgeon, Bursa Medikal Park Hospital, Hasim Iscan Caddesi Fomara Meydanı No: 1 Osmangazi Bursa, Bursa, Turkey; 2 Orthopedics and Trauma Department, Attending orthopedic Surgeon, Memorial Antalya Hospital, Zafer Mah. Yildirim Beyazit Cad. Number: 91 Kepez, Antalya, Turkey

**Keywords:** Popliteal Cyst, Baker’s Cyst, Open *excision*, Arthroscopy

## Abstract

**Background:**

Associated joint disorders with popliteal cysts were stated approximately between the ranges of 41–83 % in all reported cases. Combined treatment strategies that eliminate intra-articular pathologies and cyst- associated valve mechanisms are thought to be a good option in treatment of the disease. In this study, our main objective is to present clinical results of our combined treatment results, which includes posterior cyst excision with supine arthroscopic intervention, targeting intra-articular pathologies on recalcitrant cases.

**Methods:**

One hundred three knees of 100 patients treated with posterior open cystectomy with valve and repair of posterior capsule, in addition to arthroscopic treatment of intra-articular lesions, were included in the study. Preoperative magnetic resonance imaging (MRI) studies were performed in order to evaluate location of Baker cysts behind the knee. Rauschning-Lindgren and Lysholm Knee Scoring Scales were used to assess pre/post-operative knee functions.

Mann-Whitney *U* test was used to evaluate the differences between genders in comparison of Lysholm and Lindgren scores. Mean age within gender groups was compared using independent samples *t*-test. Wilcoxon test was used to compare the change in Lysholm and Lindgren scores. A *p*-value of less than 0.05 was considered to show a statistically significant result.

Over the 1-year follow-up period, US and MR imaging was performed only with symptomatic patients.

**Results:**

Cyst recurrence was seen only in 2 (1.94 %) patients. Post-operative Lysholm Knee and Lindgren knee scores demonstrated improvement in knee function and general comfort level of the patients.

**Conclusions:**

Our midterm follow-up (Mean: 39 Months) results showed that open cyst excision with valve and capsule repair with knee arthroscopy that targets associated intra-articular pathologies reduced the pain and improved the knee function in those patients.

**Level of evidence:**

IV (Retrospective clinical study without comparison group).

## Background

A cystic mass with large effusion within the popliteal region of the knee was first described by Guillaume Dupuytren in 1829 [1]. An association between rheumatoid arthritis and swelling of this cystic mass was subsequently reported by Robert Adams in 1840 [2]. Anatomical dissection studies identified the mass to be a distension of the bursa located between the semi-membranous tendon and the medial head of gastrocnemius muscle [3, 4]. In 1856, Foucher reported on a case of recurrent cyst, which became firm with full knee extension and softer with flexion, which was defined as “Foucher’s Sign” [5]. In 1877, Baker confirmed the entity as bursal distention, caused by trapping of fluid in a bursa with a direct relation to semi-membranous tendon. Moreover, Baker clarified that the communication between the cyst and the joint synovium behaves as a one-way valve, with fluid leaking into the bursa but with no possible flow in the reverse direction. As part of his description, Baker also described the possibility of a ruptured bursa, which resembled a venous thrombosis. Following this study, Baker’s name became associated with the clinical entity of a popliteal cyst [4, 6, 7]. Taylor and Rana also identified a communication between the medial gastrocnemius bursa and the intra-articular joint space of the knee in their 1973 postmortem dissections of 50 knees [8]. The study by Rauschning and Lindgren provided a detailed description of the association between this valve mechanism and flexion and extension movements of the knee [9, 10]. In their arthroscopic study of the anatomy of the knee joint, Kim et al. further confirmed a relationship between the presence of a capsular fold in the posteromedial capsule and the popliteal cyst [11]. Overall, associated joint disorders with popliteal cysts were reported approximately between the ranges of 41–83 % in all reported cases [12–17].

Based on the known etiology of a Baker cyst and its confirmed association with the anatomy of the knee, understanding the co-existence with intra-articular lesions, such as chondral lesions and meniscal tears that cause joint effusion and can increase pressure within the joint, and a Baker cyst is essential to clinical diagnosis and treatment. Moreover, clarifying the features of the one-way valve within the joint capsule, which leaks the effusion into the cyst, would improve our understanding of the causative factors of a Baker cyst and provide a justification for targeted treatments, including arthroscopic surgery to correct intra-articular pathologies, excision of the posterior cyst and elimination of the valve mechanism.

The use a combination of arthroscopy, performed in a supine position, targeting intra-articular pathologies and open excision of the posterior cyst in recalcitrant cases of a Baker cyst, has rarely been reported. Therefore, the aim of our study was to evaluate the clinical outcomes of treating recalcitrant cases of a Baker cyst using a combination of open-prone posterior cyst excision with arthroscopic intervention, performed in a supine position, targeting intra-articular pathologies.

## Methods

Analysis was based on the data from 103 knees, contributed by 100 patients, treated on a routine basis in our institution, using posterior open cystectomy, with valve and posterior capsule repair, and arthroscopic treatment of intra-articular lesions, between January 2009 and 2015. The study group included 40 males and 60 females, with a mean age of 49 years (range, 30–80 years) and a mean follow-up period of 37.98 months (range, 6–78) months. Patients with a previous history of knee surgery and those with other mass lesions near or adjacent to the knee were excluded.

All patients with a popliteal mass, or mass-like symptoms including pain in the popliteal fossa and/or various degrees of joint limitation consistent with physical findings of a probable Baker cyst, underwent ultrasound (USG) or magnetic resonance (MR) imaging. Patients with an identified cyst >3 cm in diameter were included (136 patients) in this study. All patients received conservative treatment for approximately 6 months prior to the decision to proceed to a surgical treatment. Conservative treatment included ice application, rest and use of non-steroidal anti-inflammatory drugs. Among our study group, improvement in symptomology was obtained with conservative treatment in 16 patients, with another 15 patients improving with use of intracystic injections. Among the remaining cases, 5 patients were lost to follow-up, with the remaining 100 patients (with 103 knees) being resistant to conservative treatment.

**Fig. 1:**
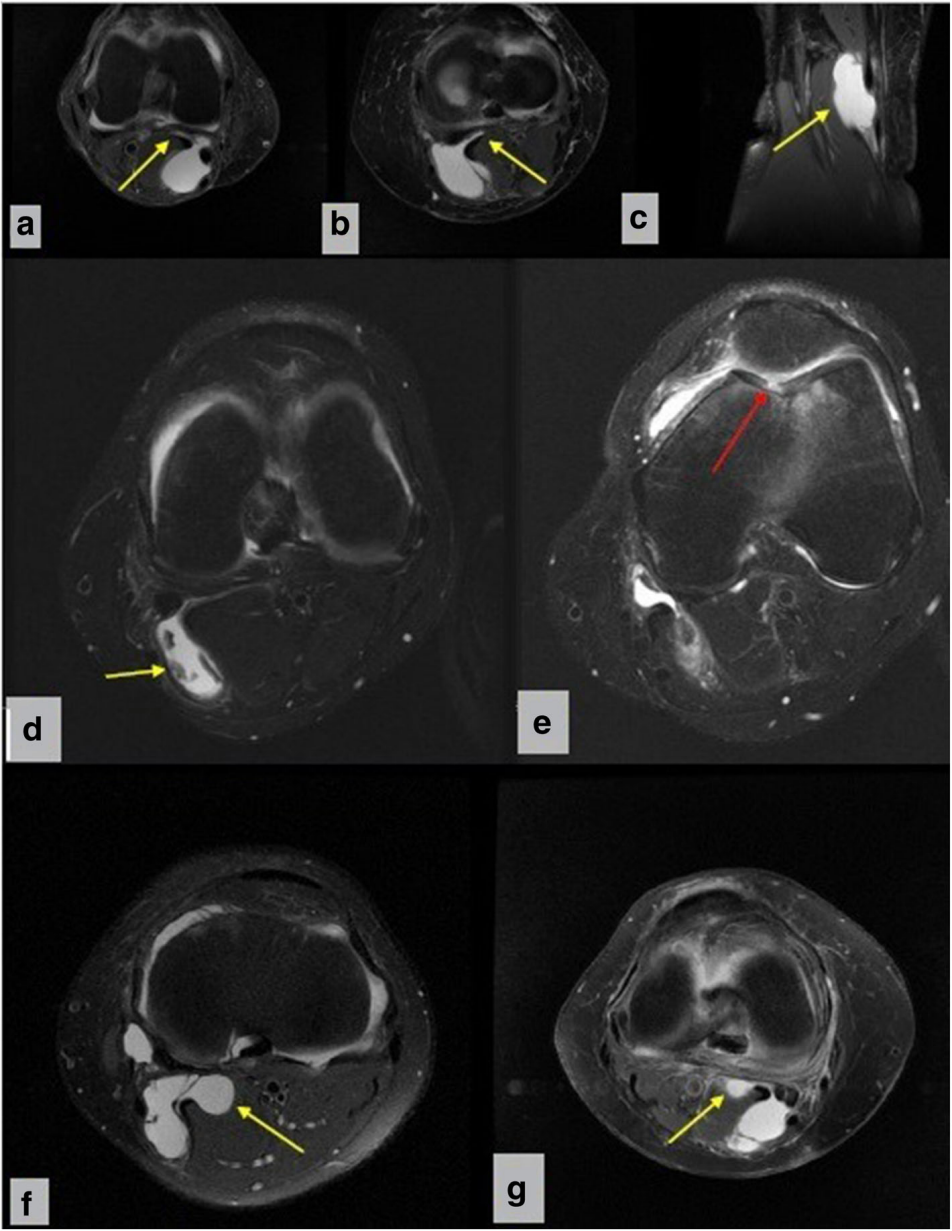
Selected sample MRI studies which demonstrate the typical peduncle and cartilage lesions. **a**, **b**- Different sample cases, axial images; notice the peduncle (*yellow arrow*) of the cyst adherent to posterior capsule. **c**- Coronal T2 weighted image that shows cyst (*yellow arrow*) located near the gastrocnemius medial head. **d**. Axial image that shows intracystic loose bodies (*yellow arrow*), originated from trochlear fissure that moved through the valve and trapped, a typical case that demonstrate the loose bodies in the cyst and effusion. **e**. The same case with figure d, The arrow (*red*) shows trochlear cartilage fissure that generate chondral debris. **f g**. Different sample cases, axial images; notice the cyst (*yellow arrow*) adherent to posterior capsule

With regards to planning of the surgical treatment, the location of the Baker cyst was confirmed from pre-operative MR images (Fig. 1 a-d, f-g), with identification of the intra-articular lesion (Fig. 1e) and/or associated knee pathologies, such as meniscal tears, chondral lesions, anterior cruciate ligament lesions, and degenerative arthritis or synovitis (Table 1). All preoperative assessments were performed by the same surgeon. The criteria for surgical treatment included a posteromedial cystic lesion (located between the gastrocnemius medial head and semimembranosus tendon), detected either on MR (Fig. 1 a-d, f, g) or US images, with a radius >3 cm, and with symptoms which are generally associated with intra-articular lesions (Fig. 1e). Surgical treatment was also recommended for popliteal cysts that recurred after aspiration, including the presence of swelling, pain or limitation in knee joint motion. The Rauschning-Lindgren Knee Score [10, 18] and the Lysholm Knee Scoring Scale were used to assess knee function before surgery and at ≥1 year post-surgery. These data are summarized in Tables 2–3.

**Table 1:** Distribution of intraarticular pathologies that were detected in pre operative MRI investigation

Pre-operative MRI imagining Findings		n	Percent
Meniscal tear (graded at MRI regrading assessed with changes in signal intensity)	Med. meniscus. Grade-1	9	8,7
	Med. meniscus Grade-2	10	9,7
	Med. meniscus Grade-3	19	18,4
	Med. meniscus Grade-4	38	36,8
	Total	74	73,6 %
	Lat. meniscus Grade-1	-	-
	Lat. meniscus Grade-2	2	1,9
	Lat. meniscus Grade-3	13	12,6
	Lat. meniscus Grade-4	5	4,9
	Total	20	19,4 %
Chondral lesion medial fem. condyle. (graded at MRI regarding to changes in signal intensity)	Grade-1	10	9,7
	Grade-2	41	39,8
	Grade-3	14	13,5
	Grade-4	4	3,8
	Total	69	66,9
Chondral lesion of Patella	Grade-1	1	0,9
	Grade-2	3	2,9
	Grade-3	9	8,7
	Grade 4	32	31
	Total	45	43,5
Plica	None	50	48,5
	İnfra	34	33,0
	Supra	19	18,4
	Total	53	51,4
Effusion	None	43	41,7
	Mild	29	28,2
	Severe	31	30,1
	Total	60	58,3
ACL rupture	Non	94	91,3
	ACL rupture	9	8,7

**Table 2:** Mean values of the patients according to sex

	Male (*n* = 43)	Female (*n* = 60)	*p*
Mean change in pre-op and post-op Lysholm scores	23.9 ± 4.2	23.3 ± 4.5	0,52*
Median change in pre-op and post-op Lindgren scores	−1,47 ± 0,55	−1,42 ± 0,56	0,52**
	−1,00 (-2,00 – (-1))	−1,00 (-1,00 – (-2))	

*Independent samples *T*-test; **Mann Whitney *U* test

**Table 3:** Mean difference in pre-op and post-op Lysholm and Lindgren Scores

	Male (*n* = 40)	Female (*n* = 60)	*p*
Age (mean ± SD)	47,60 ± 13	50 ± 10.8	0,30*
Lysholm pre-op (Median-IQR)	63.5(61.25–67)-	63 (60–65)	0.27**
Lysholm post-op (Median-IQR)	88 (84.25–92)	87 (84–90)	0.12**
Lindgren pre-op (Median-IQR)	2 (1–2)	2 (1–2)	0.33**
Lindgren post-op (Median-IQR)	0 (0–1)	0 (0–0)	0.04**

*Independent samples *T*-test; **Mann Whitney *U* test, IQR: Interquartile range

In terms of surgical treatment, all patients underwent prone posterior cystectomy and arthroscopic procedure in a supine position, including partial menisectomy, microdrilling or microfracture of chondral lesions and plica resection. Following surgery, anti-thrombo-embolic guidelines of United Kingdom National Institute for Health and Clinical Excellence (NICE) were used for all patients and thromboprohylaxis was continued for 28–35 days during the post-op period [19]. During the postoperative follow-up, only patients with symptoms underwent further US or MR imaging to assess recurrence of the cyst and/or intra-articular lesion.

For analysis, cases were categorized in terms of age, sex, type of chondral lesion and the Outerbridge classification of chondral damage, and the Rauschning-Lindgren and Lysholm Knee scores. Kolmogorov-Smirnov test was used to assess the normality of the distribution of data. Normally distributed continuous variables (demographic and baseline variables) were expressed as a mean ± standard deviation, with between-group (gender) differences (regarding to mean age) evaluated using independent samples *t*-test. Ordinal or non-normally distributed data (such as Lysholm and Rauschning-Lindgren scores) were reported by their median value and inter-quartile range. Between-sex differences regarding to pre-to-post operative scores of Lysholm and Rauschning-Lindgren tests were evaluated using a Mann-Whitney *U* test, with pre-to-postoperative change in Lysholm and Rauschning-Lindgren scores evaluated using a Wilcoxon test and independent samples *t*-test. Significance level was set as *p* < 0.05 and all analyzes were performed using SPSS 16.0 for Windows (SPSS Inc. Chicago, Illinois, USA).

## Surgical technique

### Prone open excision

**Fig. 2:**
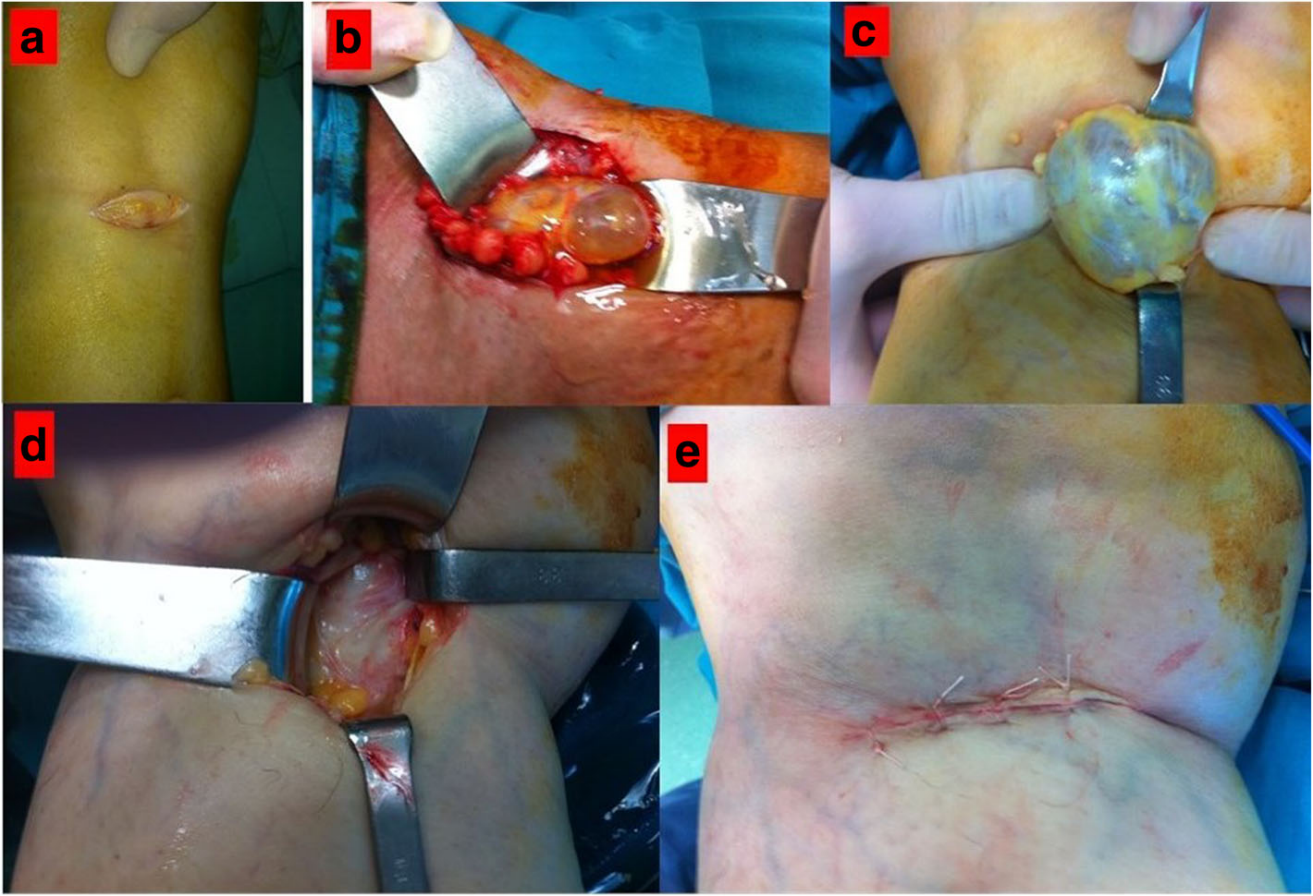
**a**-**e** Surgical technique sequence: Transverse incision parallel to flexor crease and skin lines (**a**), cyst exposure and protrusion, (**b**, **c**) posterior capsule wall following excision (**d**), the appearance following skin closure (**e**)

Patients underwent open surgery, under either general or spinal anesthesia, in a prone position, using the surgical approach and technique for cystectomy described by Wolfgang Rauschning [20], with slight modification to the skin incision and capsule closure techniques. The exposure technique used was similar to the technique described by Snir et al. [21]. A pneumatic tourniquet was applied to the upper thigh and a 5–6 cm long, transverse incision, parallel to the flexor crease and skin line, was made (Fig. 1-a). A longitudinal division of the fascia was performed to expose the semi-membranous tendon and the medial wall of the cyst. The protruding sac of the cyst was exposed (Fig. 2-b, c) and released by blunt dissection down to its line of attachment to the popliteal space. The proper plane for this dissection was evident in most cases. The interval between the semi-membranous muscle and the medial head of the gastrocnemius was opened and the wall of the cyst was separated from these structures. As no important nerves or vessels lie in this newly opened plane, dissection was continued, but with increasing difficulty in separating the cyst from adjacent structures as the dissection continued along this plane due to adherence of the cyst wall to the deeper structures. Sharp dissection was used in regions of cyst wall adherence, with fibrous components of either the semi-membranous and/or gastrocnemius muscles being included. Moreover, rupturing of a cyst further limited clear identification of the outline of the cyst, including determination of the presence or absence of a pedicle or joint communication. Since the base of the cyst is intimately attached to the capsule and synovium, any small opening connecting the cyst to the synovial cavity was located by injection of physiological saline solution with methylene blue. Following complete excision of the cyst (Fig. 2-d), the wound was copiously irrigated. The capsule was closed using No. 1 Vicryl (Ethicon, Somerville, NJ) in taut sutures. Following a second round of irrigation, the subcutaneous tissues and superficial skin were closed with tension-free sutures (Fig. 2 e).

### Supine arthroscopy

Arthroscopy, targeting treatment of intra-articular lesions, was performed in a supine position, using an anterior approach, under spinal or general anesthesia. A standardized approach using anterolateral (AL) and anteromedial (AM) portals. In selected cases, superolateral and superomedial portals were also used for additional chondroplasty under the patella. Pathological plicas were excised by shaving, and partial meniscectomy was performed for meniscal tears located within 2/3^rd^ of the central area of the meniscus. Chondral lesions with an Outerbridge classification grade of 2 and 3 were debrided by shaving. A microfracture technique was used to treat Outerbridge grade 4 chondral lesions. All excised cysts were sent to the pathology lab for histopathological examination and the nature of a cyst confirmed.

Postoperatively, patients were immobilized in 30° of knee flexion for 1 week to protect the wound from maceration or dehiscence. Based on the intra-articular treatment, patients were referred to physical therapy for standard post-arthroscopy treatment.

## Results

The distribution of age was comparable between males and females in the study group (*p* = 0.30; Table 2).

### Preoperative imaging findings

The distribution of articular pathology associated with a Baker cyst was as follows: medial meniscus tears were identified in 74 (73.6 %) knees; lateral meniscus tears in 20 knees (19.4 %); medial femoral chondral lesions in 69 knees (66.9 %); patellar chondral lesions in 45 (43.5 %); medial plica in 53 (51.4 %) knees; and ACL rupture in 9 (8.7 %). Effusion was considered mild in 29 (28.2 %) knees and major in 31 (30.1 %; Table 1).

### Functional scores

There was a significant increase in the median Lysholm score from pre-operative baseline (63.7 ± 3.5) to postoperative score (87.3 ± 4.2; *p* < 0.001, *Wilcoxon test) the mean (range) Rauschning-Lindgren score was also significantly lower after surgery (mean, 0; range, 0-1) than at preoperative baseline (mean, 2; range, 1–2; *p* < 0.001, *Wilcoxon test).

There were no between-sex differences in preoperative Rauschning-Lindgren (*p* = 0.33) or Lysholm (*p* = 0.27) scores (Table 2). Postoperative Lysholm scores were also comparable between males and females (*p* = 0.12; Table 2). However, there was a significant between-sex difference in the postoperative Lindgren score, with better functional outcomes reported by females (*p* = 0.04; Table 2). When considering the change in score from baseline (Table 3), the change in the Lysholm score (*p* = 0. 52) and the Rauschning-Lindgren (*p* = 0.52) scores was comparable for males and females, indicative of a comparable improvement in both sexes (Table 3).

### Post-operative imaging studies

Over the 1-year follow-up period, US and MR imaging was performed only with symptomatic patients. Overall, 9 patients reported discomfort and pain over the follow-up period, with 4 patients undergoing US imaging and 4 MR imaging. A recurrent cyst was identified in 2 of these patients, for an overall incidence rate of 1.94 %. There was no incidence of neurovascular injury during the surgical procedure, and no incidence of postoperative deep vein thrombosis. A superficial infection developed in 3 patients, with all 3 cases resolved following treatment with oral antibiotics.

## Discussion

We demonstrated that open posterior cystectomy, performed in combination with arthroscopic treatment of intra-articular pathologies of the knee, reduced pain, and improved symptoms and knee function. In their evaluation of the MR images of 400 knees, Miller et al. [17] identified a significant relationship between the presence of a Baker cyst, and knee joint effusion, meniscal tears and degenerative arthropathy. Specifically, they reported co-existence of a Baker cyst in 80.5 % of cases of medial meniscal tear, 31 % of cases of ACL rupture, 76.6 % of cases of knee joint effusion; and 68 % of cases of knee joint degenerative arthritis. The co-existence of a Baker cyst with a medial plica, a medial femoral chondral lesion or a lateral meniscal tear was not evaluated. A cross-sectional study published by the Arthritis Research and Therapy group reported that cartilage defects were significantly and positively associated with popliteal cysts [22].

### USG-guided aspiration with intra-articular or intracystic corticosteroid injections

With regards to treatment of Baker cysts, several studies have reported reduced cyst size and improvement in knee joint function using US-guided aspiration with intra-articular or intracystic corticosteroid injections [23–26]. However, negative results of aspiration were reported in cases with complex cyst including septation [23–26]. Overall, these studies provide evidence of very good clinical results obtained with US-guided aspiration, which has the advantage of being a non-invasive treatment technique. It is important to note, however, that these studies had relatively smaller sample sizes and short follow-up periods in comparison to our study.

### Sclerotherapy

Injection of irritating substances into cystic cavities, also known as sclerotherapy, has a long clinical history [27]. Various substances have been used for sclerotherapy of cystic cavities, including ethanol, phenol, tetracycline, 12.5 % dextrose-morrhuate solution and group-A streptococcus pyogenes [28–33]. Although positive outcomes have been reported using these methods, this evidence is based on relatively small case series studies and, therefore, higher level of evidence is required for declaring these treatments as safe and effective. More recently, fibrin glue has become increasingly popular for sclerotherapy; however, this technique does not currently have sufficient support in the literature to be recommended [34, 35].

### Arthroscopic treatment targeting intra-articular causes. *with* or *without* cystectomy, valve opening, valvectomy or valve suturing

Sasone et al. [15] treated 30 patients with a Baker cyst by arthroscopy, targeting correction of the valve mechanisms with the treatment of intra-articular lesions causing the effusion. They reported good clinical outcomes over a 2-year follow-up period. In Calvisi V et al’s [36] study done in 2007, the authors performed arthroscopy that targeted on suturing the valve (slit) with treating intra-articular lesions which cause effusion. In their case series of 22 patients followed over a 24-month period, Calvisi et al. reported cyst disappearance in 67 % of cases and a reduction in the size of the cyst in 27 % cases, with the cyst persisting in 9 % of cases. In another study done on 31 patients, Ahn JH et al. [37] performed knee arthroscopy, which targeted treatment of intra-articular lesions and resection of capsular fold through the posteromedial portal and arthroscopic cystectomy through posteromedial cystic portal for complex cysts. They reported good results in 94 % and cyst disappearance in 55 % of the patients during a mean 36-month follow-up period. Cho et al. [38] performed arthroscopic excision of a Baker cyst using posteromedial portal with arthroscopic treatment of associated intraarticular lesions in 111 patients, they reported no recurrence of the cyst over a 24-month follow-up period. In their case series of 16 patients, Rupp et al. [39] reported unsuccessful outcomes, over a 24-month follow-up period, for treatment targeting solely the intra-articular lesion, without consideration for the cyst and its valve mechanism. In their treatment of 14 patients with a large Bake cyst (>5 cm), Ko et al. [40] reported successful outcomes over a 12-month follow-up period using debridement by cystoscopy with excision of the capsular fold and valve mechanism. Based on their experience, Ohishi et al. [41] recommended use of an additional posterior trans-septal portal to improve visualization of the posteromedial compartment of the knee. In their study, Ohishi et al. performed arthroscopic cyst decompression via enlargement of the unidirectional valve slit through two posterior portals, without resection of the inner wall of the cyst. They reported successful results in 85.9 % of the 29 patients forming their cases series, with cyst disappearance identified in 75.9 % of cases and size reduction in 10 % of cases over an average follow-up of 22.9 months. Lie and Ng reported good-to-excellent outcomes among their 11 patients treated by anterior arthroscopy for an intra-articular derangement over a 13-month follow-up period [42]. In this study, Lie and Ng established posteromedial portal to resect valve openings and a trans-cystic portal for debridement of the septum in cases of multiple septa within the cyst.

A technical note by Takahashi and Nagano [43] introduce an arthroscopic technique using posteromedial and posterolateral portals to treat a popliteal cyst by disrupting the slit-like structure. This technique also provides excellent arthroscopic visualization of the cavity of the popliteal cyst through the knee joint approach. Regarding to establishment of the posterior portals they used Ahn and Ha’s [44] method.

Kongmalai et al. [45] described the use of a direct arthroscopic posterior portal in addition to a posteromedial portal for overcoming difficulties in intracystic debridement of cysts with septation.

In summary, the arthroscopic techniques described above could be useful for the treatment of intra-articular pathologies. However, using posteromedial and posterior portals for targeted treatment of the valve and cyst requires experience and surgical skill. Moreover, due to the technical difficulty of the procedure, total cyst wall excision cannot be achieved in all cases.

### Open excision

During the 1970’s, Childress [13] underlined the association between medial meniscus tears and popliteal cysts. Based on his 22 years of experience, treating 215 cases of Baker cysts, Childress recommended treatment using an anterior arthrotomy for treatment of intra-articular pathologies and posterior open-cyst excision, with reinforcement of the posterior capsule by suturing both the medial head of gastrocnemius and tendon of the semimembranosus muscles to the capsule over the defect. Evaluation of 84 consecutive cases of popliteal cyst excisions, cyst recurrence was identified in 3 cases (3.5 %). Rauschning and Lindgren [18] performed an open posterior excision of a Baker cyst in 46 knees, using 6 different types of incisions. In 25 of their cases, the site of communication between the cyst and the intra-articular joint space (also referred to as the pedicle) was successfully closed, with closure either not attempted or not successful in the remaining 16 cases. In our study, all communications (valves/capsular folds) were closed. Another difference between Rauschning and Lindgren’s study and ours was that they used catgut suture material for capsule repair, with a reported 51.2 % rate of cyst recurrence. Poor visual field for cyst exposure, difficulty in closing the valve communication and the weak tension strength of catgut suture are likely factors contributing to the poor clinical outcomes over the 48-month follow-up of the 40 patients in their study. In our study, we used 1/Vicryl sutures, which is much stronger than catgut. Moreover, since we eliminated the factors causing effusion by targeting intra-articular pathologies, the effusion, which increased the pressure against the tensile strength of the sutures, was decreased.

Rauschning et al. [20] modified their original technique by including a partial gastrocnemius-pedicle graft to reinforce the capsular repair. With this modification, they reported no recurrence of the cyst or post-operative complication. However, this follow-up study included only 8 patients and did not target intra-articular pathologies. Hughston et al. [46] described a similar surgical approach in their case series of 24 patients (25 knees), reporting excellent outcomes in 20 of the 25 knees (80 %), with 2 cases of cyst recurrence. Fair results were reported in another 3 knees, with 2 ultimately being treated with total knee replacement due to degenerative arthritis. Again, the cohort in this study was small and intra-articular pathologies were not targeted in the arthroscopic treatment, with an overall recurrence rate of the Baker cyst of 8 %.

### Arthroscopic treatment, targeted on intra-articular causes, combined with posterior open excision

Snir et al. [20] described the open exposure for posterior excision of a Baker cyst following arthroscopic medial meniscectomy in one patient. They described a detailed open exposure, which was similar to our exposure methods in detail, we were able to identify the pedicle and perform valve closure and capsule repair, easily, in all patients in our case series with this exposure.

### General comments

It is now well-established that formation of Baker cysts is associated with intra-articular pathologies of the knee, and in particular with medial meniscus tears or other conditions that cause general effusion of the knee. The classic paper by Wolfgang Rauschning [9] provides a thorough description, with illustration, of the etiopathogenesis of popliteal (Baker) cysts, including the presence of capsular openings (communication link) between the gastrocnemius-semimembranosus bursa and the knee joint. This slit-shaped capsular orifice behaves as a valve, which opens during knee flexion due to pulling force of semimembranosus tendon and closes with extension due to compressing forces of overlying tendons. The anatomical relationship between popliteal cysts and the posteromedial capsule was explored using arthroscopy by Kim et al. [11]. They reported a statistically significant relationship between the presence of a capsular fold, with and without an opening, and the formation of a Baker cyst. Therefore, when present, a capsular fold and its opening should be completely resected, and the remnant capsule repaired using strong suturing materials, which can resist pressure changes that occur during knee flexion-extension movements. We postulate that the low recurrence rates is that we targeted intra-articular pathologies with cyst excision and capsule repair using strong absorbable suturing materials (Vicryl/1). We agree with the idea that treating intra-articular pathologies and performing closure of the valve, in addition to cyst excision, is beneficial and provided evidence of successful clinical outcomes of this combined approach in our case series. In comparison to our study, Cho et al. [38] treated 111 patients using a combined treatment consisting of anterior arthroscopy for intra-articular pathologies and cyst excision using a posteromedial portal, with patients followed for a period of 24 months post-surgery. This study did not have same features with our study with shorter follow-up period.

In contrast to our approach, Childress [13] combined posterior cyst excision with an anterior arthrotomy to treat intra-articular lesions, which significantly requires prolonged the period of recovery, before the age of arthroscopy.

Therefore, after performing an extensive review of the literature, we did not identify any study, other than those of Cho et al. and Childress [13, 38], reporting on the outcomes of a combined strategy of anterior arthroscopy and open posterior cystectomy in a large cohort followed over a sufficient period post-surgery. We do note that our study is also limited by its retrospective design and absence of a control group.

## Conclusions

We provided evidence that open cyst excision, with valve and capsule repair, performed with the patient in a prone position, and combined with knee arthroscopy performed in a supine position for treatment of intra-articular pathologies, is a safe and reliable treatment model in treatment of Baker cysts.

## Abbreviations

Mm: Millimeter
MRI: Magnetic resonance imaging
US: Ultrasound
